# Premature aging in telomerase-deficient zebrafish

**Published:** 2013-09

Premature aging is a feature of the genetic disorder dyskeratosis congenita (DC). DC is caused by progressive shortening of telomeres, sequences that protect chromosome ends. Telomere shortening occurs in the normal process of aging, and mutations in telomerase, the enzyme that maintains telomere length, can accelerate this process. Although mouse models have provided insight into the role of telomeres in premature aging, they do not recapitulate all human symptoms. Here, María Cayuela’s group show that telomerase-deficient zebrafish demonstrate the hallmark features of premature aging in the first generation, in contrast with mice, which have to be bred for several generations. Intriguingly, the zebrafish mutants show ‘anticipation’ (onset of disease at a younger age in the next generation), which is also apparent in individuals with DC. Reintroduction of telomerase is able to rescue telomere length, confirming the importance of telomerase dosage and paving the way for studies of new therapeutics based on telomerase reactivation. **Page 1101**

